# Microbial Seafloor Weathering of Hydrothermal Sulfides: Insights from an 18-Month In Situ Incubation at the Wocan-1 Hydrothermal Field

**DOI:** 10.3390/biology14040389

**Published:** 2025-04-09

**Authors:** Chuanqi Dong, Xiqiu Han, Yejian Wang, Jiqiang Liu, Mingcong Wei

**Affiliations:** 1 College of Marine Geosciences, Ocean University of China, Qingdao 266100, China; cqdong@sio.org.cn; 2 State Key Laboratory of Submarine Geoscience, Second Institute of Oceanography, Ministry of Natural Resources, Hangzhou 310012, China; yjwang@sio.org.cn (Y.W.); liujq@sio.org.cn (J.L.); 9120230010@jxust.edu.cn (M.W.); 3 Ocean College, Zhejiang University, Zhoushan 316021, China; 4 School of Oceanography, Shanghai Jiao Tong University, Shanghai 200240, China; 5 Jiangxi Provincial Key Laboratory of Low-Carbon Processing and Utilization of Strategic Metal Mineral Resources, Ganzhou 341000, China

**Keywords:** hydrothermal sulfides, in situ incubation, microbial weathering, Carlsberg Ridge, Northwest Indian Ocean

## Abstract

The weathering of seafloor hydrothermal sulfides is facilitated by microbial activities, yet the specific mechanisms of different sulfide types are not well understood. Previous studies have primarily been carried out under laboratory conditions, making it difficult to accurately replicate the complex in situ conditions of deep-sea hydrothermal fields. Herein, we deployed two well-characterized pyrite (Py)-dominated and chalcopyrite (Ccp)-dominated sulfide slices, which were placed 300 m from an active venting site in the Wocan-1 hydrothermal field (Carlsberg Ridge, Northwest Indian Ocean) for an 18-month in situ incubation experiment. Microscopic observations and organic matter analyses were conducted on the recovered sulfide slices to investigate the microbial weathering features of different sulfide types. Our results demonstrated that the weathering of the Py-dominated sulfide sample was primarily mediated by extracellular polymeric substances (EPSs) through indirect interactions, whereas the Ccp-dominated sulfide sample exhibited both direct microbial dissolution, resulting in the formation of distinct dissolution pits, and indirect EPS-mediated interactions. Four distinct phases of microbe–sulfide interactions were identified: approach, adsorption, stable attachment, and extensive colonization. Furthermore, the weathering products and biomineralization structures differed significantly between the two sulfide types, reflecting their different microbial colonization processes. Our study confirms that microorganisms are crucial in seafloor sulfide weathering. These findings advance our understanding of microbial-driven processes in sulfide mineral transformations and their role in marine ecosystems. Our findings are also valuable for future research on biogeochemical cycles and for developing bioremediation strategies for deep-sea mining.

## Simple Summary

The weathering of seafloor hydrothermal sulfides is mediated by microorganisms. To investigate their weathering characteristics and mechanisms, we conducted an 18-month in situ incubation experiment using two well-characterized hydrothermal sulfide slices placed 300 m from an active venting site in the Wocan-1 hydrothermal field. Our findings revealed that the weathering of chalcopyrite-dominated sulfides occurred via direct microbial dissolution, whereas the weathering of pyrite-dominated sulfides relied on indirect interactions that were mediated by microbial extracellular polymeric substances. Distinct biomineralization patterns were observed that indicated sequential colonization phases, including approach, adsorption, attachment, and colonization. These findings provide valuable insights into the microbe–mineral dynamics found in extreme environments, highlighting the significant roles of microbes in seafloor sulfide weathering. This knowledge advances our understanding of marine biogeochemical cycles and offers a foundation for developing sustainable bioremediation strategies for deep-sea mining.

## 1. Introduction

Submarine hydrothermal systems play a vital role in the exchange of energy and matter among the hydrosphere, lithosphere, and biosphere. They host abundant polymetallic sulfides, which are important mineral resources and have the potential for future exploitation [1]. The seafloor weathering of these sulfide minerals is of significant interest owing to its potential impact on both their economic potential and marine biogeochemical cycles [2,3]. This weathering process involves two synergistic mechanisms: chemical weathering and microbial weathering. Chemical weathering is predominantly driven by abiotic reactions such as oxidation and hydrolysis. Microbial weathering is driven by microbial metabolic activities, including Fe^2+^/S^0^ oxidation and EPS-mediated mineral dissolution [2,3]. The former process is governed by environmental parameters (e.g., temperature, pH, and ionic concentration), whereas the latter is driven through direct microbial–mineral surface interactions or indirect interactions mediated by metabolic byproducts (e.g., Fe^3+^ and organic acids) [4,5]. Among these mechanisms, microbial weathering, driven by sulfur-oxidizing bacteria (SOB) and iron-oxidizing bacteria (FeOB), is especially significant. It not only accelerates mineral transformation but also directly influences the bioavailability of essential metals and sulfur compounds, thereby playing a critical role in shaping the biogeochemical cycles and ecological functions of marine environments [4,5].

While significant progress has been made in understanding microbial processes in seafloor hydrothermal systems, research on the weathering of polymetallic sulfides, particularly the microbial mechanisms involved and their interactions with these minerals, remains limited [6,7,8,9]. Most previous studies have been conducted under controlled laboratory conditions, which fail to replicate the complex and dynamic in situ environments of deep-sea hydrothermal systems. Consequently, the mineralogical and morphological changes induced by microbial weathering in these extreme environments are still poorly understood. Addressing this knowledge gap is critical, especially as interest grows in polymetallic sulfides as targets for deep-sea mining, a practice that may profoundly impact marine ecosystems [6,7].

Py and Ccp are two major hydrothermal sulfide minerals [8,9]. However, the microbial processes involved in their weathering remain incompletely characterized. The properties of Py and Ccp influence their susceptibility to microbial weathering [10,11]. The crystal structure of pyrite, which is composed of iron and sulfur atoms, belongs to the isometric crystal system, exhibiting symmetrical forms such as cubes and octahedrons, which are prone to disruption during microbial weathering processes [6,12]. Pyrite’s higher surface reactivity provides favorable conditions for microbial attachment and oxidative reactions [5]. Research indicates that pyrite is more susceptible to microbial weathering under acidic, oxidizing, or hydrothermal conditions while remaining relatively stable in neutral or low-temperature environments [13]. A previous study by Kocaman et al. (2016) demonstrated that *Acidithiobacillus ferrooxidans* can oxidize iron in pyrite, leading to ferrous ion release and sulfate formation, thereby accelerating pyrite dissolution [13]. In contrast, chalcopyrite with primary crystallization in the tetragonal system exhibits greater resistance to microbial weathering under certain conditions [5]. The presence of copper in chalcopyrite’s crystal structure can inhibit microbial growth and activity [14]. However, oxidation products from chalcopyrite, such as copper ions, may enhance the dissolution of other minerals. Zhang et al. (2021) revealed that *Acidithiobacillus ferrooxidans* oxidizes the iron in chalcopyrite, resulting in copper ion release and sulfate generation [15]. The microbial weathering of chalcopyrite typically requires specialized strategies like biofilm formation and indirect oxidation pathways, potentially slowing its weathering rate [13].

Pyrite demonstrates slower microbial weathering rates in cold/neutral conditions but becomes highly reactive in acidic, oxidizing, or hydrothermal systems [13]. Comparatively, chalcopyrite shows moderate reactivity in neutral environments but faces kinetic limitations and passivation layers (comprising materials such as jarosite, elemental sulfur, and iron oxyhydroxides) in acidic or hydrothermal systems unless exposed to elevated temperatures or iron ion cycling [15]. These differences indicate that the susceptibility of pyrite and chalcopyrite depends not only on their crystal structures and chemical compositions but also on microbial preferences and environmental settings.

Léveillé and Juniper (2002) [16] conducted the first long-term (12-month) in situ weathering experiment in the hydrothermal field of the Juan de Fuca Ridge, using slices of massive sulfide minerals. Lipid analyses confirmed the presence of sulfate-reducing bacteria (SRB) and filamentous SOB in the enriched samples. Edwards et al. (2003a) [8] performed in situ incubation experiments using various metal sulfides on the Juan de Fuca Ridge. Their results revealed extensive microbial weathering of hydrothermal sulfide samples, accompanied by significant aggregates of the secondary iron oxides associated with FeOB on weathered surfaces. In addition, the density of bacterial colonization followed a specific order, from highest to lowest: elemental sulfur > chimney sulfide > marcasite > Py > sphalerite > Ccp. This pioneering study continues to provide valuable insights into the function and distribution of bacteria colonizing natural sulfide minerals. Subsequent in situ incubation experiments identified SOB (*γ*- and *δ-Proteobacteria*) and FeOB (*γ*- and *ζ-Proteobacteria*) as the dominant species colonizing mineral surfaces. These studies highlight the bioavailability of sulfide minerals as a key factor in shaping microbial populations [17,18,19,20]. Microbe-mediated sulfide weathering releases bioavailable metals (e.g., Fe^2+^, Cu^2+^) and sulfate, thereby modifying local chemical gradients in deep-sea environments and subsequently regulating chemoautotrophic ecosystem biogeochemistry [6]. Consequently, the quantitative assessment of weathering dynamics has become imperative for predicting the long-term ecological perturbations associated with deep-sea mining operations. However, previous research has primarily focused on microbial diversity, community composition, and bacterial abundance across different sulfide types. Detailed descriptions of microbial mineralization structures and their relationships to sulfide oxidation mechanisms remain scarce. Additionally, comparative analyses of in situ oxidation processes among the various sulfides are limited. Further investigations are needed to elucidate the specific mechanisms driving microbial oxidation, particularly regarding modes of contact and the verification of weathering processes.

In this study, we conducted an 18-month in situ incubation experiment with (Py)-dominated and (Ccp)-dominated massive sulfide samples, which had been placed 300 m from the Wocan-1 hydrothermal vent on the Carlsberg Ridge. Using a combination of microscopic and spectroscopic techniques, we explored the mechanisms of the microbially driven weathering of these minerals. This approach allows for a comprehensive understanding of how microorganisms interact with sulfide minerals.

## 2. Materials and Methods

### 2.1. Sample Selection

**Figure 1:**
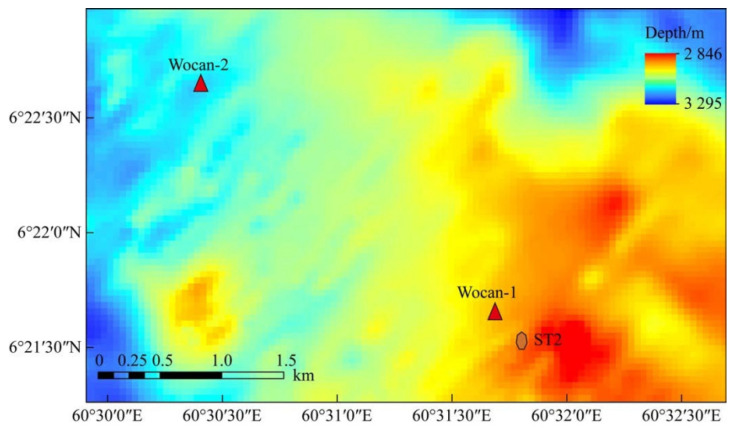
Bathymetric map of the Wocan-1 hydrothermal field, showing the location of the sediment trap.

Sulfide samples were collected from the Wocan-1 hydrothermal field (6°22′ N, 60°31′ E, at a water depth of 3120 m), which is a typical mafic-hosted, high-temperature hydrothermal system located on the Carlsberg Ridge in the Northwest Indian Ocean (Figure 1) [21,22]. There are two types of sulfide samples: Py-dominated and Ccp-dominated samples. The Py-dominated samples were sliced directly, whereas the Ccp-dominated samples were first embedded in epoxy resin to prevent fragmentation. All samples were cut into thin slices, measuring approximately 5 cm × 3 cm, using a diamond saw.

**Figure 2:**
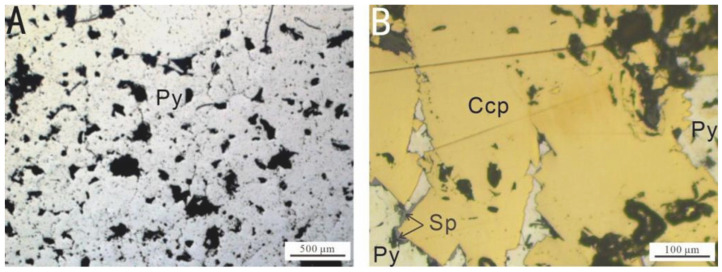
Microscopic images of the surfaces of the samples selected for the in situ incubation experiment at the Wocan-1 hydrothermal field: (**A**) Py-dominated samples; (**B**) Ccp-dominated samples. Abbreviations: Py, pyrite; Sp, sphalerite; Ccp, chalcopyrite.

The mineral composition of the sulfide samples is presented in Table 1. Before the experiment, the samples were examined using an optical microscope (Figure 2) to determine their mineral composition, texture, and morphology. The mineral slices were subsequently cleaned by immersion in anhydrous ethanol for 2 h, followed by ultrasonic cleaning for 10 min to remove potential contaminants [8].

### 2.2. In Situ Incubation Experiments on the Seafloor

**Figure 3:**
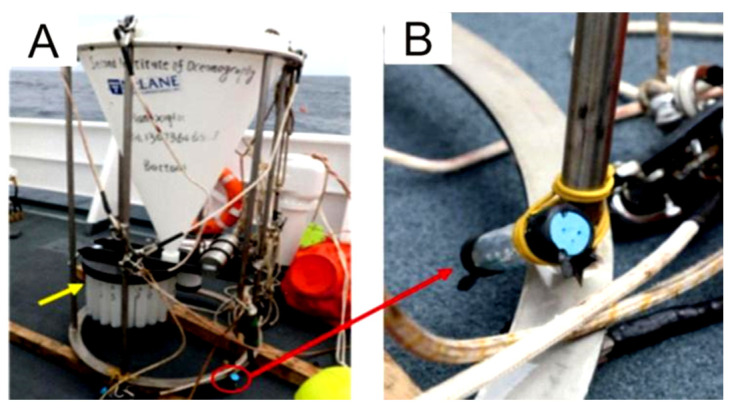
Sediment trap equipped with an in situ incubation device. (**A**) The recovered sediment trap remains intact. The red circle marks the location where the bottle containing the sulfided samples was fixed for in situ incubation. An enlarged view of the device is shown in (**B**).

PVC bottles, fitted with numerous holes with a diameter of 3 mm, were prepared to contain the sulfide samples for the experiment. The bottles were then secured to the leg of a sediment trap, approximately 10 cm above its foot (Figure 3). The sediment trap, along with a mooring system, was deployed in June 2018, during the DY49-5 cruise in the Wocan-1 hydrothermal field, within approximately 300 m of the active venting site (Figure 2, ST2). The device was retrieved in June 2019 during the DY57 cruise, achieving a total seafloor in situ incubation duration of 18 months. Upon recovery, the samples were immediately stored in a refrigerator at 4 °C.

### 2.3. Cell Staining and Microstructure Analysis

The recovered sulfide slices were rinsed with ultrapure water, freeze-dried, and cut into smaller pieces. The LIVE/DEAD^®^ BacLight™ Bacterial Viability Kit (Thermo Fisher, Waltham, MA, USA), which contains SYTO 9 and propidium iodide components, was used to stain the bacterial cells [23]. After staining, the slices were placed on sterile slides, and two to three drops of mounting medium (Thermo Fisher) were applied to the surface. The samples were then visualized using appropriate excitation and emission filters to distinguish the fluorescence signals of live and dead bacteria under a fluorescence microscope (Eclipse 80i, Nikon, Tokyo, Japan).

The stereomicroscope (Leica EZ4 W, Leica Camera AG, Wetzlar, Germany) was used to identify the differences in the samples’ surfaces before and after in situ incubation. Microstructural and elemental analyses were performed with a Phenom XL G2 benchtop high-resolution scanning electron microscope (SEM) and energy-dispersive spectroscopy (EDS) at Funa Scientific Instruments (Shanghai) Co., Ltd. (Shanghai, China).

### 2.4. FT-IR Analysis

The FT-IR analysis of the samples was conducted at the Second Institute of Oceanography, Ministry of Natural Resources, Hangzhou, China, using a Nicolet 6700 FT-IR spectrometer (Thermo Fisher, USA). The analysis was performed with a KBr beam splitter and a liquid nitrogen-cooled MCT-A probe, at a resolution of 4 cm^−1^ and a wave number range of 600–4000 cm^−1^, with non-polarized polarization and 128 scans. The analysis was conducted at room temperature (20 °C), with a humidity level of 30–40%. A background value was deducted for each sample analyzed. The infrared spectral data obtained were processed using the OMNIC 7.3 software, with transformations applied to transmittance and absorbance, image smoothing, and baseline calibration. Infrared spectral peaks were deconvoluted using Gaussian peak fitting in Origin software, and the areas of the fitted peaks were calculated. The baseline calibration of FT-IR spectra was performed using the automatic baseline correction module in OMNIC 7.3 to eliminate instrumental background interference. Gaussian peak deconvolution prioritized the identification of characteristic absorption bands (e.g., C=O stretching and P=O vibration), with the subsequent optimization of peak area quantitation through iterative least-squares minimization. Overlapping peaks were resolved via FWHM (full width at half-maximum) constraints during spectral deconvolution [24]. The processed data were then exported and plotted using Origin 2022.

## 3. Results

### 3.1. Surface Morphology Changes Before and After the Incubation Experiment

**Figure 4:**
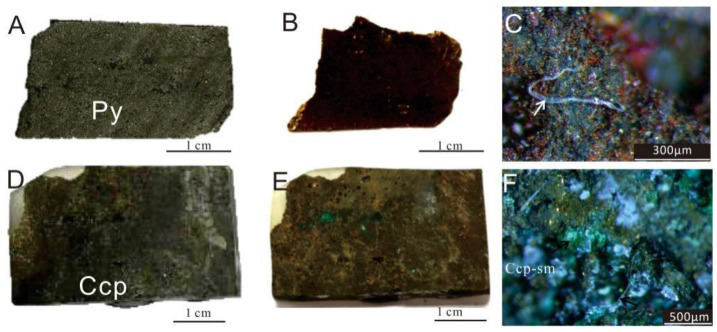
Images of sulfide samples before and after in situ incubation experiments. (**A**) Py-dominated sulfide slice before the experiment. (**B**) Py-dominated sulfide slice after the experiment. (**C**) Py-dominated sulfide slice after the experiment, seen under a stereoscopic microscope, showing the presence of brownish-red iron oxides (Fe-ox), observed filamentous structures resembling biogenic origins (white arrow). (**D**) Ccp-dominated sulfide slice before the experiment. (**E**) Ccp-dominated sulfide slice after the experiment. (**F**) Ccp-dominated sulfide slice after the experiment, seen under a stereoscopic microscope, showing the presence of greenish secondary minerals (sm).

The surfaces of the sulfide samples exhibited significant differences before and after the experiment (Figure 4). A distinct brownish-red layer, composed of oxides and secondary minerals, was newly formed (Figure 4B,D). The blue-green secondary weathering products of Cu-sulfides (covellite, malachite, etc.) were observed on the surfaces of Ccp-dominated samples, especially within the voids or pits (Figure 4F).

### 3.2. Observations Under SEM

#### 3.2.1. Oxides Formed During the Incubation Experiment

**Figure 5:**
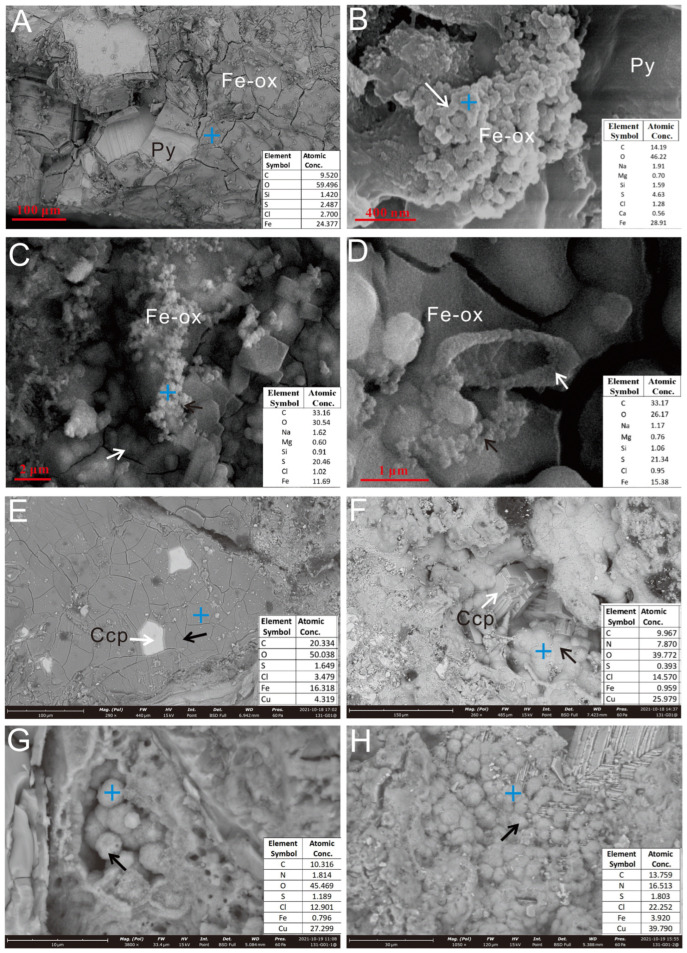
SEM images of Py-dominated (**A**–**D**) and Ccp-dominated sulfide samples (**E**,**F**) after in situ incubation. (**A**) Pyrite grains were coated with a thin layer of iron oxides. (**B**) The sheath-like structure, composed of iron oxides (white arrow). (**C**,**D**) Aggregates of iron oxide particles (white & black arrow). (**E**) Charcopyrite grains (white arrow), coated with a thin layer of iron oxides (black arrow). (**F**) Partial transformation of Ccp crystals (white arrow) into secondary Cu oxides (black arrow). (**G**) Spherical secondary Cu oxide particles (black arrow) grew inside the dissolution hole. (**H**) Partial oxidation of Ccp crystals, forming secondary Cu oxide aggregates (black arrow). The blue cross icon indicates the EDS spectrum location, hereinafter the same.

SEM analysis showed that, following incubation, the surface of the Py-dominated sulfide sample underwent significant oxidation and was coated with a thin layer of Fe oxide, approximately 5–10 μm in thickness (Figure 5A). Nano-sized iron oxide globules were observed in the pores, forming clusters and aggregates (Figure 5B–D). The Ccp-dominated sulfide sample also underwent significant oxidation, developing a 2–3 μm thick iron oxide layer on its surface (Figure 5E). Within the fractures and pores were aggregations of Cu oxide globules, with sizes ranging from 0.1 to 5 μm (Figure 5F–H).

#### 3.2.2. Microbial Mineralization Structures

**Figure 6:**
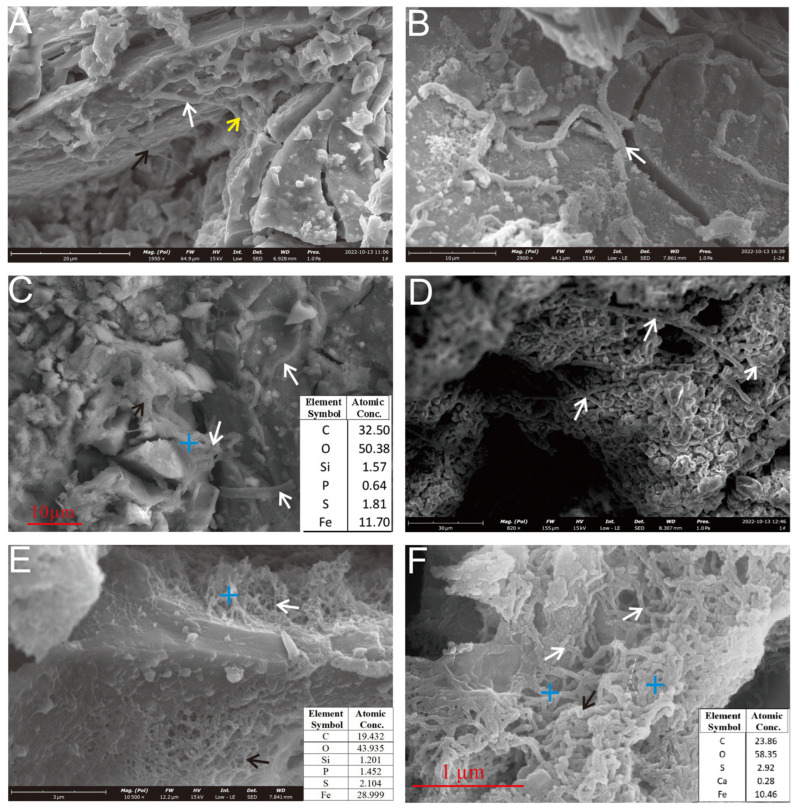
SEM observation of the microbial mineralization structures of a Py-dominated sample. (**A**) Mineralized microbial filaments on the sample surface face exhibit a tapering trend at the ends (indicated by arrows). (**B**) Fine filaments aggregate into larger, curved, and coiled filaments (white arrow). (**C**) The filaments are intertwined (black arrow) and a hollow tubular structure is visible (white arrows). (**D**) Rod-like sheaths, typically formed by iron-oxidizing bacteria such as *Leptothrix ochracea* or *Mariprofundus ferrooxidans*, are interspersed between the oxide particles (white arrows). (**E**) Rod-like sheaths (white arrow) and curved stalks (black arrow) form reticulated aggregates. (**F**) Curved filaments (white arrows) and twisted filaments (black arrow) aggregate to form a reticulated structure.

SEM analysis revealed numerous curved stalks and filamentous structures on the surface of the oxidized layer of the Py-dominated sample (Figure 6). These mineralized stalks and filaments, typically measuring 20–30 μm in length and approximately 1 μm in diameter, primarily consist of iron (hydro)oxides. Based on surface morphology, the filaments can be categorized into two distinct types: rough-surfaced and smooth-surfaced.

The rough-surfaced filaments present a hair-like appearance, with curved and coiled morphologies. These structures likely originate from the aggregation of numerous finer, hair-like subunits (Figure 6B). In contrast, smooth-surfaced filaments are commonly associated with extracellular polymeric substances (EPSs) (Figure 6A). Occasionally, filaments with tapered tips and variable diameters can be observed (Figure 6A, black and yellow arrows). The morphological features of these filaments, particularly the twisted forms and EPS associations, closely resemble those produced by the iron-oxidizing bacterium (FeOB) *Mariprofundus ferrooxidans* (*Zetaproteobacteria*) [10,25,26,27,28,29,30].

Further analysis of the pyrite (Py) surfaces revealed two additional morphotypes: curvilinear filaments and straight rod-like sheaths (Figure 6C–F). They are associated with EPSs. The curved stalks (Figure 6F, black arrow) were intertwined into reticulated networks, analogous to the skeletonized microtextures generated by *Acidithiobacillus ferrooxidans*-mediated dissolution [11]. The straight rod-like sheaths were dispersed among the oxide particles (Figure 6D, white arrow). These types of morphology are commonly associated with those of FeOB, such as with *Leptothrix ochracea* or *Mariprofundus ferrooxidans* and *Zetaproteobacteria* [10,31,32]. Notably, the reticulated networks likely reflect localized microbial activity driving intense sulfide dissolution, contrasting with the biofilm-associated filaments found on stable sulfide substrates.

**Figure 7:**
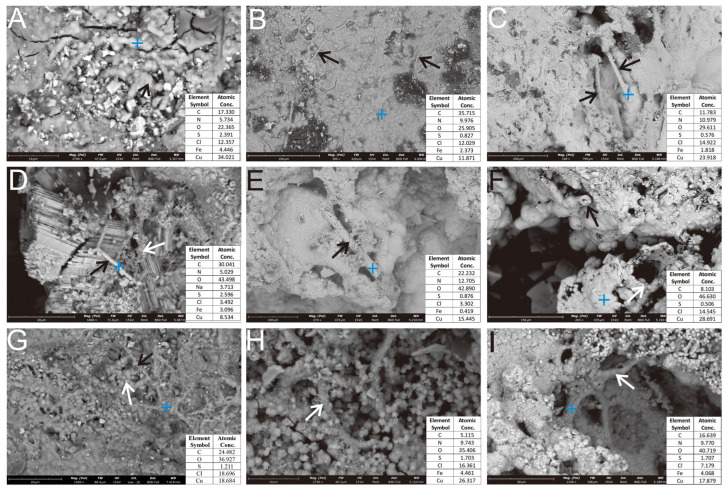
SEM micrograph of filamentous and stalk-like structures in the Ccp-dominated sample. (**A**) Rod-like structures, arranged in a concentric circular pattern (black arrow). (**B**) Elongated stalk-like structures, attached to the surface of the oxide layer (black arrows). (**C**) Rod-like structures developed within the dissolution pits (black arrows). (**D**) Rod-like structures (black arrow), associated with spherical nanoparticles (white arrow). (**E**) Hollow tubular morphology, formed by aggregated oxidation products (black arrow). (**F**) Secondary Cu minerals, coalescing to form a hollow tubular structure (black & white arrow). (**G**) Primary sulfide surfaces, coated with globular secondary Cu minerals (white arrow) and sheathed mineralized filaments (black arrow). (**H**) Curved stalk surfaces, adsorbed with numerous globular secondary Fe and Cu mineral particles (white arrow). (**I**) Hollow tubular sheaths, composed of aggregated nanocrystalline secondary Fe and Cu minerals developing inside a dissolution pit (white arrow).

SEM imaging of the Ccp-dominated sulfide sample showed stalk-like structures, predominantly composed of the oxidation products of Ccp. Key observations included a smooth, multi-ringed, annular rod (~1 μm in diameter), interconnected by spherical particles (Figure 7A, black arrow). Elongated stalk-like structures (2–5 μm in diameter, up to 100 μm in length) were attached to the surface of the oxidized layer (Figure 7B, black arrow), morphologically analogous to the biogenic structures produced by *Leptothrix ochracea* or *Mariprofundus ferrooxidans* [31,33,34]. Coarse rod-like structures (15–20 μm diameter) within the dissolution voids (Figure 7C, black arrow) were found alongside smaller and rougher-surfaced rods (~2 μm in diameter), interspersed with numerous fine-grained Fe and Cu oxides (Figure 7D, white arrow). Hollow tubular structures (~15 μm in diameter) were formed by the aggregated oxidation products, exhibiting loose to dense textural gradients (Figure 7E, black arrow). Cross-sections of the filaments revealed the central lumens (Figure 7F, black arrow). Larger tubular aggregates (60 μm in diameter) resulted from the stacking of smooth secondary mineral particles (1–20 μm in diameter; Figure 7F, white arrow). Curved stalks (~2 μm in diameter) were adsorbed with globular secondary mineral particles (0.5–2 μm in diameter; Figure 7H, white arrow), and the hollow tubular sheath (5–8 μm in diameter), which was composed of aggregates of tiny secondary mineral particles, was visible in the pits (Figure 7I, white arrow).

#### 3.2.3. Microbial Cells and EPSs

**Figure 8:**
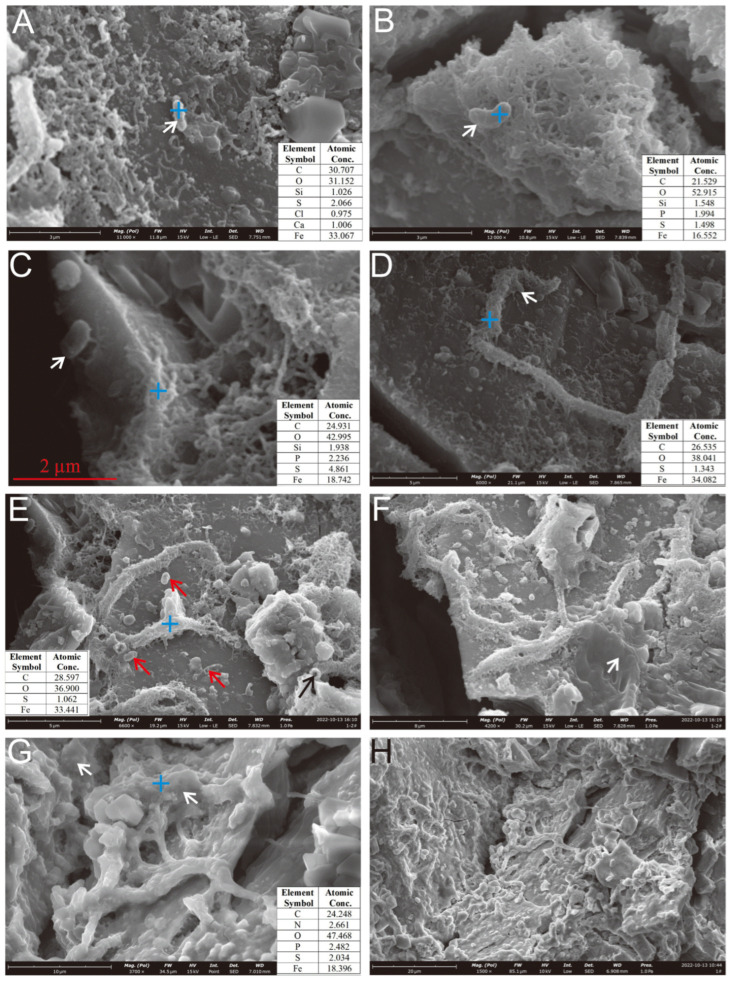
Microbial cells and EPSs on the surface of the Py-dominated sample. (**A**) Cellular morphology structures developing near HFO filaments and granular aggregates (white arrow). (**B**) Cellular structures above the hollow reticulated HFO aggregates (white arrow). (**C**) Cellular morphology structures on the surface of pyrite crystals (white arrow). (**D**) Cellular morphology structures near the curved, rod-shaped mineralized HFOs formed by filament aggregates (white arrow). (**E**) Mineralized filaments accumulate to form curved tubes with thicker bodies; “bean-shaped” cells are present on the weathered Py crystal surfaces (red arrows), visible hollow tubular mineralization (black arrow). (**F**–**H**) The surface of the oxidized pyrite is extensively coated with a substantial layer of EPSs (white arrows).

**Figure 9:**
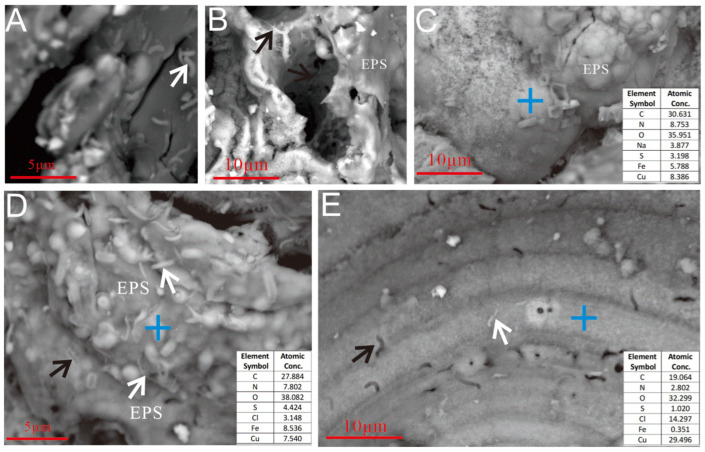
Microbial cells and EPSs on the surface of the Ccp-dominated sample. (**A**) Microbial cells are distributed on the surface of the sulfide, 1–3 μm in length and 0.2–0.5 μm in diameter (white arrow). (**B**) Microbial cells are found on the surface of the oxidation products of sulfide and within the dissolution cavities (black arrows). (**C**) The surface of the sample was covered by a layer of EPSs. (**D**) Globular mineralized structures, 1~2 μm in diameter, are visible on the surface of the Ccp matrix and are covered with an EPS layer. Numerous microbial cells, 0.5~3 μm in length and 0.1~0.4 μm in diameter, are present on the surface of the matrix (white arrows), along with pits formed by microbial erosion (black arrow). (**E**) Microbial cells on the surface of the Ccp matrix (white arrow) and the cellular morphology of erosion pits (black arrow).

SEM imaging revealed FeOB-like microtextures on weathered sulfides (Figure 8 and Figure 9). Bacterial cells and their metabolic products (e.g., EPSs and iron oxides) exhibited a close spatial relationship with etch pits (Figure 8C), providing direct evidence of active microbe-mineral interactions during seafloor sulfide weathering. The EPSs, which are rich in organic groups such as carboxyl (-COOH) and phosphoryl (PO_3_^2−^), provided negatively charged sites that facilitated cation chemisorption. These chemically bonded metal ions acted as nucleation centers, promoting further complexation and mineralization [35,36].

On the weathered surface of the Py-dominated sample, cell-like structures resembling *Thiobacillus ferrooxidans* [37] were observed, anchored via organic bridging networks (Figure 8C). *Acidithiobacillus ferrooxidans*-like cells, an iron-oxidizing bacterium widely studied in laboratory settings [11], were found in association with aggregates of hydrous ferric oxides (HFOs) on the oxidized surface (Figure 8A) and within curved, rod-shaped mineralized HFOs formed by filament coalescence (Figure 8D,E). Similar structures were also identified in hollow reticulated HFO aggregates (Figure 8B). The characteristics of the secondary mineral aggregates also resembled those formed by *Acidithiobacillus ferrooxidans* [11]. Additionally, the Py-dominated sample was covered by EPS layers on its curved filament aggregates (Figure 8F) and curved rod surfaces (Figure 8G,H).

Regarding the weathered surface of Ccp-dominated samples, microbial cells were found attached to the oxidation product layer, either directly adhering to the sulfide surface (Figure 9B,D,E, white arrow) or residing within the cavities (Figure 9C). Distinct dissolution pits, resembling the morphology of microbial cells, were also observable on the sulfide surface (Figure 9D, black arrow; Figure 9E, black arrow). A layered EPS was observed to be forming on the oxidation product surface surrounding the microbial cells (Figure 9B–D).

#### 3.2.4. Cell Staining and Fluorescence Microscopy Observation

**Figure 10:**
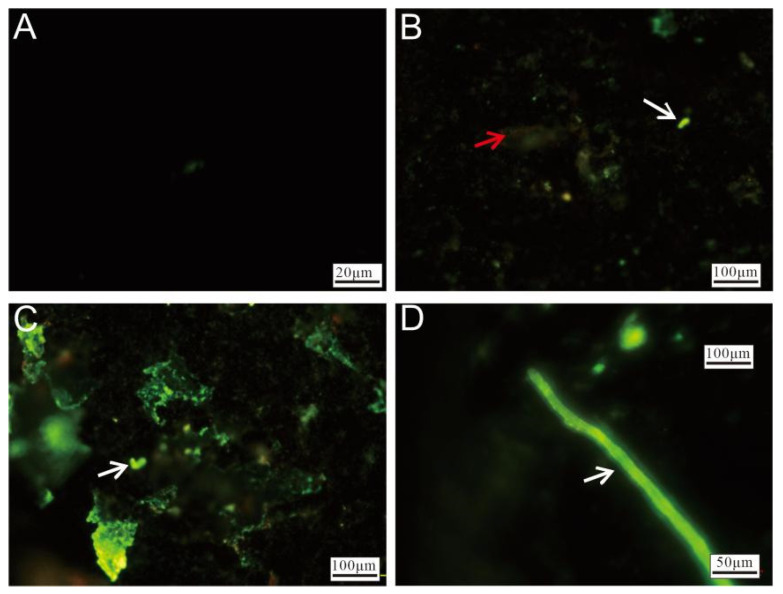
Fluorescence microscopy images of Py-dominated samples after staining. (**A**) Fluorescence microscopy image of unstained pyrite. (**B**) Weathered minerals (red arrow) and microbial cells (white arrow), highlighted by staining. (**C**) Surface of the pyrite, showing extensive coverage by nucleic acid-rich materials, with microbial-like cellular structures visible (white arrow). (**D**) Microbial-origin rod-like structures (white arrow).

Fluorescence microscopy of the Py-dominated samples revealed significant differences between unstained (Figure 10A) and stained (Figure 10B–D) sulfide slices after incubation. The stained slices exhibited varying intensities of green fluorescence, with red fluorescence being observed in certain regions. The red fluorescence (Figure 10B) primarily originated from oxidized minerals. The brighter green fluorescence displayed morphological characteristics resembling microbial cells (Figure 10B,C, white arrow), indicating microbial colonization. Areas with intense, widespread green fluorescence suggested that the surfaces of the sulfide slices were likely covered with nucleic acid-containing substances, implying the substantial attachment of cell membranes or EPSs. Additionally, rod-like structures, likely formed through microbial activity, were also observed (Figure 10D).

**Figure 11:**
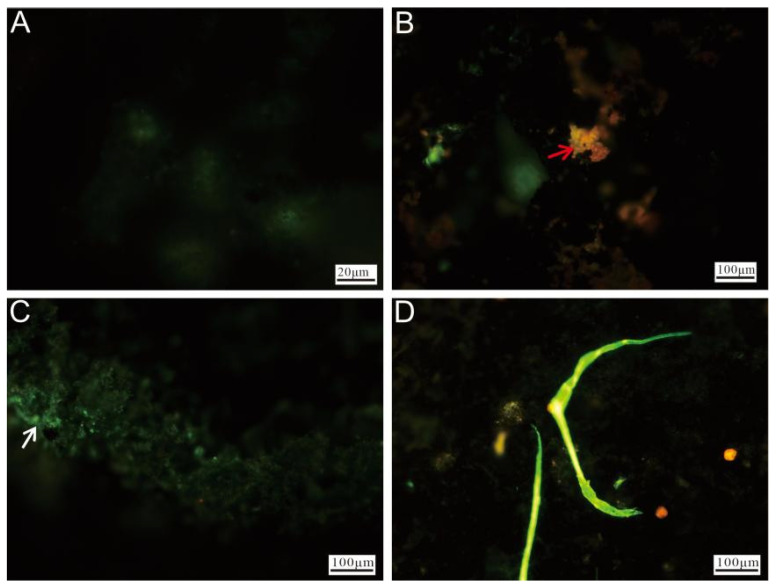
Fluorescence microscopy images of Ccp-dominated samples after staining. (**A**) Unstained chalcopyrite, showing minimal fluorescence. (**B**) Weathered minerals after staining (red arrow). (**C**) The surface of chalcopyrite, covered by nucleic acid-rich material (white arrow). (**D**) Filamentous structures formed by microbial activity.

Fluorescence microscopy of Ccp-dominated samples revealed that the unstained regions exhibited minimal fluorescence (Figure 11A). The red fluorescence observed in the stained areas (Figure 11B) primarily originated from the oxidized minerals. A distinct green fluorescence, likely associated with the microbial cells, was also visible on the left side (Figure 11C). Additionally, prominent biomineralized filamentous structures were observed, representing stable features formed during prolonged biomineralization processes (Figure 11D).

### 3.3. FT-IR Analysis of Organic Functional Groups

#### 3.3.1. Organic Functional Groups in Sulfides Before and After Experimentation

**Figure 12:**
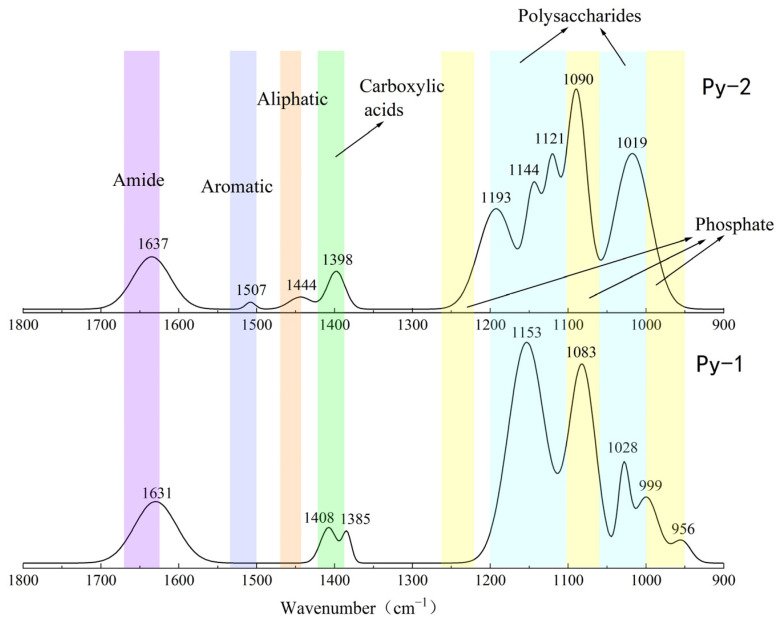
Comparison of the FT-IR analysis results of Py-dominated samples before (Py-1) and after (Py-2) in situ incubation.

**Figure 13:**
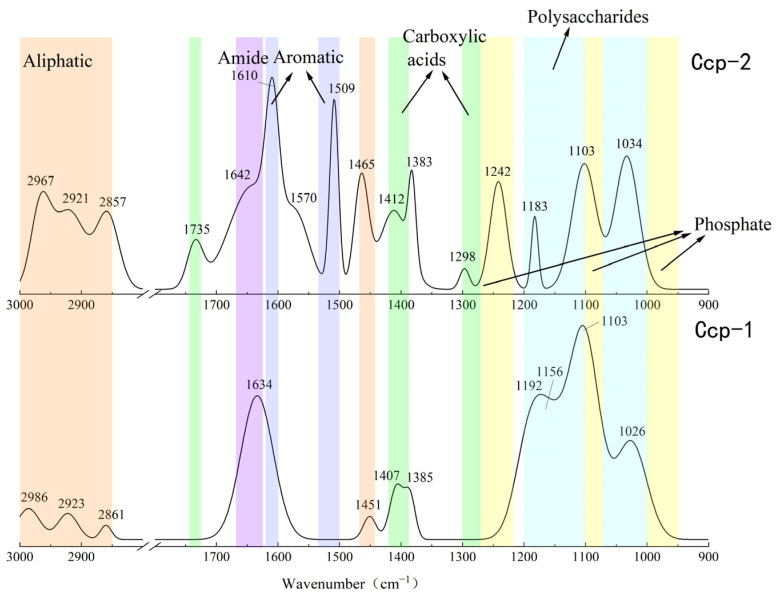
Comparison of the FT-IR analysis results of Ccp-dominated samples before (Ccp-1) and after (Ccp-2) in situ incubation.

The results of the FT-IR analysis for the sulfide samples before and after the seafloor in situ incubation experiments are shown in Figure 12 and Figure 13. Significant changes in the functional group peaks were observed.

The FT-IR analysis of the sulfide samples, both before and after in situ incubation, identified six major classes of organic functional groups: aliphatic compounds, carboxylic acids, amides, aromatic compounds, phosphate groups, and polysaccharides. The principal FT-IR absorption bands are summarized in Table 2.

The FT-IR analysis of the Py-dominated slices after in situ incubation revealed new spectral peaks at 1507 cm^−1^, 1444 cm^−1^, 1193 cm^−1^, and 1121 cm^−1^. The peak at 1444 cm^−1^ corresponds to the C-N stretching vibration in the amide group, while the peak at 1398 cm^−1^ is attributed to the symmetric stretching vibration of the carboxyl group (-COO-) [23]. Amides are primarily associated with proteins, while carboxylic acids are linked to proteins, fatty acids, and uronic acids [44].

Before the in situ experiment, the peak at 1083 cm^−1^ corresponded to the asymmetric stretching vibration of PO^2−^ [45]. After the in situ experiment, the peak at 1090 cm^−1^ was attributed to the asymmetric stretching vibration of FeO-P-OFe [44]. This shift in phosphate group absorption indicates that the phosphate groups in nucleic acids within the EPSs can exchange with hydroxyl groups on the surface of acicular ferrite, forming P-OFe bonds [44,46]. This implies that the adsorption of nucleic acids plays a significant role in EPSs binding to iron oxide surfaces.

For the Ccp-dominated samples, FT-IR analysis identified peaks at 2967 cm^−1^, 2921 cm^−1^, 2857 cm^−1^, and 1465 cm^−1^, indicating the presence of lipids containing the CH_2_ groups [45]. The peaks at 1735 cm^−1^, 1412 cm^−1^, 1383 cm^−1^, and 1298 cm^−1^ suggest carboxylic substances containing the C=O functional groups [37,38]. The peak at 1642 cm^−1^ corresponds to the C=O stretching vibration of amide II in proteins [44]. Peaks at 1610 cm^−1^ and 1509 cm^−1^ are indicative of aromatic compounds containing C=C bonds, specifically suggesting the presence of tryptophan [47]. The peak at 1242 cm^−1^ can be attributed to the P=O group in nucleic acids (DNA and RNA) or phosphorylated proteins, corresponding to the PO^2−^ group in phosphates. The peak at 1183 cm^−1^ represents C-O stretching vibrations in nucleic acids, while the peak at 1103 cm^−1^ corresponds to P-O asymmetric stretching in phosphates [48]. Finally, the peak at 1034 cm^−1^ is associated with polysaccharides [23].

#### 3.3.2. Organic Fractions in Sulfides Before and After In Situ Incubation

**Figure 14:**
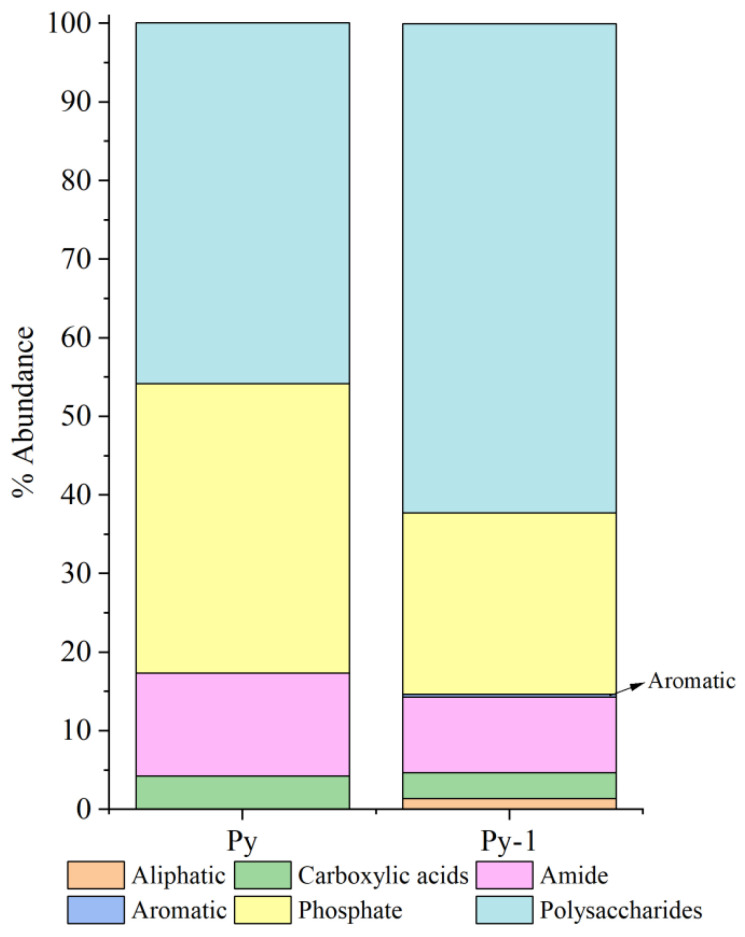
Semi-quantitative analysis of the abundance of organic components based on FT-IR peaks, fitting for Py-dominated samples.

**Figure 15:**
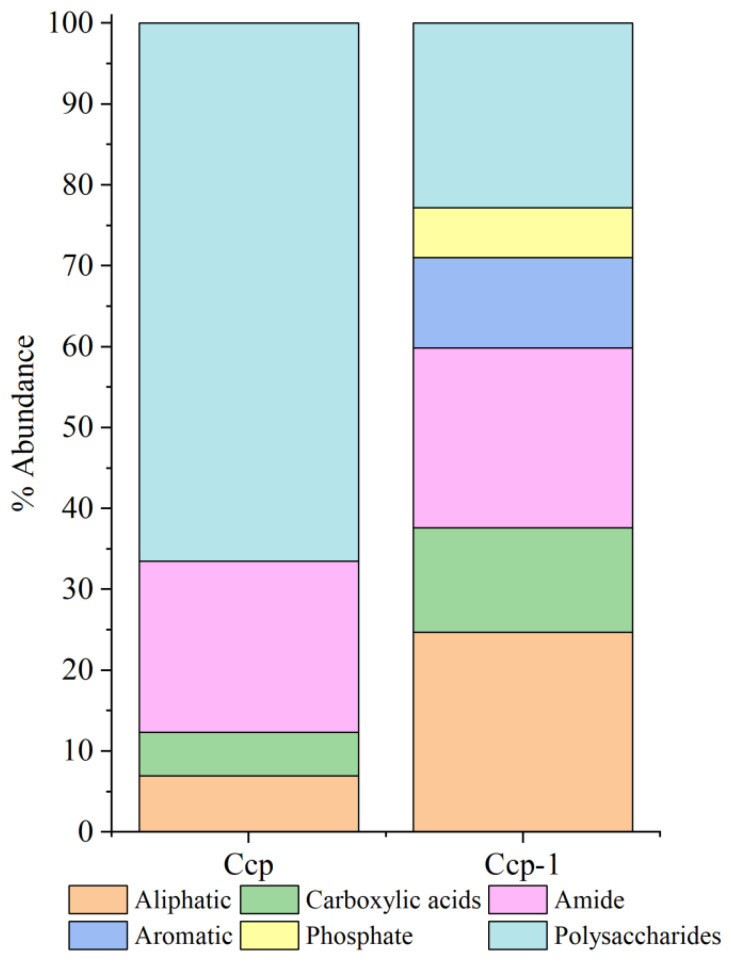
Semi-quantitative analysis of the abundance of organic components based on the FT-IR peaks, fitting for Ccp-dominated samples.

Relative peak intensity is defined as the percentage of the area of a specific characteristic peak, relative to the total area of all characteristic peaks. It reflects the relative abundance of the corresponding functional group within the sample [38,49]. By fitting and calculating the peak areas of organic functional groups before and after the in situ incubation of the sulfide samples, we observed significant changes in the relative peak areas of each functional group (Figure 14 and Figure 15). After the in situ experiment, aliphatic and aromatic organic matter in the Py-dominated samples accounted for 1.37% and 0.34% of the total characteristic peak area, respectively. Notably, the proportion of polysaccharides increased by 16.31%. In the Ccp-dominated samples, following in situ incubation, the proportions of aliphatic, carboxylic acids, aromatic, and phosphate compounds increased by 17.75%, 7.59%, 11.21%, and 6.12%, respectively, demonstrating a significant rise (Table 3).

## 4. Discussion

### 4.1. Characterizing Microbial Interactions with Sulfide Minerals

#### 4.1.1. Surface Evidence of Microbial Weathering

Our results suggest that the substantial accumulation of alteration products on sulfide surfaces primarily results from in situ oxidative dissolution, rather than from the deposition of particles that are present in the surrounding water. Previous research has demonstrated that chemolithoautotrophic microorganisms can mediate the weathering and dissolution of sulfide minerals, leaving distinctive microbial imprints on mineral surfaces. A prominent visual feature of this process is the formation of dissolution pits on sulfide mineral substrates following microbe–mineral interactions. For example, Verati et al. (1999) [50] documented dissolution pits on iron sulfide substrates in hydrothermal chimney sulfide oxidation layers from the Pito seamounts, attributing them to the activity of *Acidithiobacillus ferrooxidans* and other FeOB, which drive the bioerosion of iron sulfides. These microbial imprints differ from abiotic chemical erosion patterns [51,52].

The cellular morphology of the pits observed on Ccp-dominated samples aligns with laboratory findings and differs from abiotic chemical erosion patterns [51,52]. The correlation between microbial cells (Figure 9B–E, white arrow) and cellular-structured dissolution pits (Figure 9D,E, black arrow) on Ccp-dominated mineral surfaces provides direct evidence of bacterial–mineral interactions. Microbe-mediated dissolution pits (Figure 9D,E) exhibit regular cellular morphologies, distinct from inorganic acid corrosion patterns [51,52]. Furthermore, the presence of EPSs (Figure 8F) and mineralized structures linked to specific microbial taxa, such as *Zetaproteobacteria* (Figure 6D), further supports a biogenic origin [53,54].

Previously, we performed high-throughput sequencing of the 16S rRNA genes in the samples after incubation [55]. The dominant bacterial taxa included *α*- and *γ-amorphobacteria*, *Campylobacteria*, *Anaplasma phagocytophilum*, *Cyanobacteria*, *Desulfobulbia*, and *Actinomycetes*, with the SOB *Sulfurimonadaceae*, *Thiotrichaceae*, and *Thimomicrospiraceae* being the dominant taxa. In particular, Sulfurimonadaceae can oxidize sulfides to release sulfate and lower the local pH, thereby accelerating mineral dissolution [17], whereas the EPSs secreted by *α*- and *γ-amorphobacteria*, along with their formation of iron oxide sheaths (Figure 6 and Figure 8), are closely associated with the indirect weathering mechanisms of Py [33]. Additionally, potential bacterial strains associated with the weathering of polymetallic sulfides were also observed through enrichment and isolation experiments.

In our experimental observations, an approximately 10-μm-thick iron (hydro)oxide layer was visible on the basal surface of Py (Figure 5A). Iron oxide nanospheres aggregated into clusters on the oxidized layer’s surface (Figure 5C, black arrow). The morphological features of these clusters aligned with the structure of iron oxide shells formed by the seafloor microbial oxidation of sulfide [12,56], suggesting that they represent the initial stage of oxide formation during the microbial oxidation of pyrite. Adjacent to these nanoscale sphere clusters, numerous smooth ellipsoidal iron oxide particles were observed (Figure 5C, white arrow), with diameters ranging from approximately 0.5 to 1.5 μm. These ellipsoidal particles are the aggregates of nanoparticles that originated from the initial nanoscale sphere clusters and further developed through crystallization. Over time, these nanoparticle aggregates formed tight interconnections, ultimately leading to the formation of a shell-like oxide layer (Figure 6D). The progression from nanoscale clusters to ellipsoidal aggregates, and finally to a consolidated oxide layer, illustrates the stepwise mineralogical transformation that is driven by microbial pyrite oxidation.

#### 4.1.2. Biogenic Mineralization Structures in Microbial Weathering

Structural observations of the weathered and altered sulfide surfaces revealed the presence of twisted or branched stalks, tubular sheaths, and filamentous structures. These structures, which are typically composed of iron oxides and EPSs, are recognized as reliable biosignatures of microbially mediated iron oxidation [25,57]. The distinctive morphology aligns with known FeOB, such as *Mariprofundus ferrooxydans*, *Gallionella ferruginea*, and *Leptothrix ochracea*.

The rod-shaped iron oxide ultrastructures that are newly formed by FeOB typically exhibit relatively smooth surfaces [25] (Figure 6C, white arrow). In contrast, iron oxides that form over longer periods often incorporate bacterial sheaths, stalks (Figure 6D), and filaments (Figure 7G,H) [58], showing heavier iron oxide encrustation compared to more recent formations. Poorly crystalline phases, such as two-line ferrihydrite, often co-precipitate with bacteria [58], later transforming into crystalline phases such as hematite and goethite through crystallization [59,60].

The primary type of FeOB found in seafloor hydrothermal sulfide deposits is *Zetaproteobacteria* [18,25,59,61,62,63,64,65]. The most extensively studied species, *Mariprofundus ferrooxydans*, exhibits “bean-shaped” cells and secretes organic-encased ferrihydrite stalks as metabolic byproducts [65]. These stalks can subsequently act as substrates for further ferric hydroxide precipitation, thereby increasing the overall Fe/C ratio of the previously formed stalks and making them thicker, while also masking some of their earlier characteristic features, such as twists and helical structures [25,65]. *Mariprofundus ferrooxydans* cells eventually detach from the stalks and become motile, allowing them to form new stalks elsewhere [65]. The stalks formed by *Mariprofundus ferrooxydans* range in width from 0.6 to 2.2 µm and may branch during cell division. The stalks are typically aligned in parallel as a result of coordinated growth along a chemical gradient [30,64]. In our observations, distinct “bean-shaped” cells were visible on the weathered pyrite crystal surfaces, with widths ranging from approximately 0.5 to 1 µm (Figure 8B,C,E). On our samples, extensive filamentous iron oxide aggregates formed during microbial weathering, eventually developing into thicker, curved rod-like structures (approximately 1 µm in diameter) (Figure 8E). These rod-like structures were further covered with ferric hydroxide precipitation, increasing their diameter to approximately 1.5 µm and resulting in a smoother surface that obscured the original filamentous aggregation structure (Figure 8F). This process represents a typical developmental pathway for biomineralized structures formed by *Mariprofundus ferrooxydans*. In addition, the pyrite-dominated sample was extensively covered by biofilms and EPSs. The filamentous or rod-shaped mineralized filaments were also more abundant compared to those on Ccp-dominated samples. This may be due to the increased adsorption of microbial cells onto the mineral surface in environments with higher Fe^2+^ ion concentrations, enhancing adhesion strength and thereby promoting biofilm development and accelerating the surface erosion of metal sulfides [66]. On the basis of the structural changes observed on the Py-dominated sample surface, we speculate that during the microbial weathering of seafloor sulfides, biomineralization structures undergo a growth process that begins with filamentous forms, followed by the aggregation of these filamentous forms into rough, curved rod-like structures. These structures then progress to surface coverage with organic matter, ultimately resulting in the formation of smooth, sheath-like curved structures.

The core of microbial weathering lies in the metabolic activities of microorganisms, which release energy by oxidizing iron or the sulfur in sulfide minerals. This process accelerates the mineral surfaces’ dissolution and promotes weathering product formation [33,67]. In this study, the nanoscale spherical clusters, ellipsoidal particles, and shell-like oxide layers observed on the Py-dominated sample are likely the result of the microbial oxidation of Fe^2+^ to Fe^3+^, followed by precipitation [33]. Microbial metabolic activities also regulate the chemical conditions of mineral dissolution. For example, SOB oxidizes sulfide to produce sulfate ions, thereby lowering the local pH and accelerating mineral dissolution [33,68]. The microbial oxidation of pyrite can acidify the surrounding fluids, while polysaccharide-dominated extracellular polymeric substances (EPSs) formed during bacterial leaching help buffer the surface acidity [20,69]. This aligns with the significant increase in polysaccharide organic components observed in the weathered Py-dominated samples in our study (Figure 14). Additionally, oxidation shells formed through microbial weathering inhibit further pyrite dissolution while providing substrates for subsequent ecosystems [70]. In contrast, Ccp-dominated samples exhibit cell-like morphologies and adjacent dissolution pits (Figure 9D,E), indicating microbial erosion via direct contact [71]. This process is driven by the chemical carriers secreted by the bacteria to dissolve the mineral. Such microbial activity continues efficiently, leading to the sustained erosion of Ccp-dominated sulfides.

The hydrochemical gradients (e.g., temperature, dissolved oxygen, and Fe^2+^ concentration) in the Wocan-1 hydrothermal field critically regulate microbial activity and weathering rates. The in situ experimental site, which is located ~300 m from the active venting sites, is likely within the diffusive zone of the Wocan-1 hydrothermal field. This setting provides a moderate-temperature environment that is conducive to colonization by FeOB and SOB [8]. Compared to high-temperature vents, lower-temperature zones slow chemical oxidation kinetics, enabling microbial metabolism to dominate the weathering processes [20]. Furthermore, the elevated Cu content in copper-containing minerals (e.g., Ccp) could suppress specific microbial populations via metal toxicity. However, acidophiles such as *Acidithiobacillus* spp. maintain erosive activity by secreting chelators to mitigate metal stress [39].

The rapid oxidative dissolution of chalcopyrite may induce the preferential early-stage release of metals such as copper, potentially exerting toxic effects on the benthic biota, whereas pyrite weathering exhibits prolonged dissolution kinetics through EPS-mediated passivation layer formation. This mechanism enables sustained Fe release, thereby supporting long-term chemolithotrophic community maintenance [70]. Therefore, we infer that the microbial weathering of Py-dominated and Ccp-dominated sulfides in the seafloor environment differentially modulates elemental cycling. These findings provide a foundational framework for evaluating the environmental impact of microbial weathering on various sulfide deposits during deep-sea mining extractions.

### 4.2. Stages and Mechanisms of Microbial Weathering in Hydrothermal Sulfides

Previous simulation experiments have demonstrated that metal sulfides in nature undergo distinct changes during microbial weathering. A number of studies using controlled microbial weathering simulations have observed different dissolution patterns across the various metal sulfides [53,54,72]. Zhu et al. (2014) [53] and Li et al. (2016) [54], in their studies on the oxidative dissolution of arsenopyrite and pyrite by *Acidithiobacillus ferrooxidans*, proposed a three-stage model, beginning with (1) the inorganic oxidation-dominated stage. In this stage, microbes rapidly proliferate by oxidizing Fe^2+^ ions in the solution. The resulting Fe^3+^ ions further oxidize the sulfur on the metal sulfide surface, leading to minor surface dissolution of the mineral. This stage exceeds the duration of the microbial stationary growth phase. (2) Microbe-induced mineral dissolution and biofilm formation, where microbial cells gradually adhere to the metal sulfide surface, eventually developing into a biofilm while also forming etch pits and minor secondary precipitates on the mineral surface. This stage is relatively brief, corresponding to the biofilm development cycle. (3) The co-oxidation stage, involving microorganisms and Fe^3+^ ions. In this stage, the attached microbes intensively corrode the surface of the metal sulfides, creating numerous dissolution pits that increase the contact area between the solution and the mineral, thereby promoting further mineral dissolution [69,73]. This stage is characterized by abundant secondary precipitates and significant surface dissolution pits, representing the primary phase of the microbial oxidative dissolution of metal sulfides.

After microbial weathering, nanoscale spherical iron oxide aggregates (Figure 8A) and numerous curved, sheath-like structures (Figure 8F,G) were observed on the surface of the primary pyrite substrate. A common feature of these mineralized structures is the clear presence of an EPS layer covering their surfaces, corresponding to stage (2), characterized by extensive biofilm formation and with a small amount of granular secondary mineral precipitates attached to the substrate surface (Figure 8F). This is a prominent characteristic of the initial stage of microbe–mineral interaction. In contrast, after microbial weathering of the Ccp-dominated samples, numerous microbial cells were directly attached to the Ccp substrate, accompanied by dissolution-induced pits (Figure 9D,E). Additionally, numerous spherical iron oxide precipitates formed on the Ccp surface (Figure 9D,E), with extensive EPSs covering both the microbial cells and the Ccp surface. These biomineralization features are consistent with stage (3), indicating that microbe–mineral interactions have progressed to a relatively intense phase.

In the indirect mechanism of microbial sulfide weathering, microbes generate Fe^3+^ by oxidizing Fe^2+^, which is produced through chemical reactions in the weathering solution; this Fe^3+^ subsequently participates in the weathering of metal sulfides. Notably, this process does not involve direct contact between the microbes and the mineral [74]. Contact mechanisms are categorized into direct and indirect types. The direct contact mechanism entails the microbial oxidation of minerals through biological processes, independent of ferrous or ferric ions. In contrast, the indirect contact mechanism suggests that during metal sulfide weathering, microbes secrete EPSs that create a suitable micro-niche, promoting cell adhesion to the mineral surface. In this process, the microbes oxidize Fe^2+^ to Fe^3+^ and release it extracellularly, while the sulfur within the metal sulfide is oxidized to sulfoxy species, which eventually separate from the metal sulfide surface [5,50,75]. In the synergistic mechanism, freely suspended microbes in the solution and adsorbed cells on the mineral surface work in concert: the former acts as an electron transfer pathway, transferring electrons from the reduced sulfur and Fe^2+^ to O_2_, while the latter directly participates in the dissolution of the metal sulfide [5,75].

In our experimental observations, EPSs were commonly found covering the Py substrate on the surface of the Py-dominated samples (Figure 8F,G,H), including those on curved stalk surfaces (Figure 8G) and curved filament aggregates (Figure 8F). Additionally, microbial cells were observed, attached to mineral surfaces covered with EPSs (Figure 8C), indicating that this process aligns with a typical indirect contact mechanism. In the Py-dominated samples, the dissolution pits formed by direct microbial erosion of the sulfide surface were rarely observed. Thus, we infer that in the neutral environment of the seafloor, microbe–mineral interactions involving Py predominantly occur via an indirect contact mechanism, rather than via a direct contact mechanism.

On the surface of the Ccp-dominated samples, numerous microbial cells were clearly visible (Figure 9A,D,E, white arrow), along with dissolution pits that mirror the morphology of the microbial cells (Figure 9D,E, black arrow). These observations provide compelling evidence for the direct involvement of microbes in mineral surface alteration. Rojas-Chapana and Tributsch (2000) [76], through the observation of pit formation on sulfide mineral surfaces, concluded that these pits result from the mineral dissolution caused by the chemical carriers secreted by bacteria. In our study, the surface of the Ccp-dominated samples prominently displayed cell-like structures and dissolution pits shaped like cells (Figure 9D,E). This further supports the hypothesis that the microbial metabolic erosion of sulfides occurs through direct contact. The shape of these dissolution pits is influenced by the contours and spatial distribution of cells, indicating that direct contact mechanisms represent a common mode of microbial interaction with chalcopyrite minerals. These pits closely resemble those formed during microbially mediated sulfide oxidation in laboratory settings [77,78,79]. Similar dissolution pits have also been observed in ancient sedimentary rocks [80], suggesting that such dissolution features can be preserved intact in ancient rocks for hundreds of millions of years and later identified using standard geological techniques. Therefore, these characteristic dissolution pits can serve as effective biosignatures for detecting the microbial weathering of iron-bearing minerals in both modern and ancient sediments and may even represent potential signs of extraterrestrial life. Furthermore, on the surface of the Ccp-dominated samples, after Ccp weathering, EPSs were observed covering the oxidized layer (Figure 9C) and spherical iron oxide aggregates on the Ccp surfaces (Figure 9C). EPSs coexisted with rod-shaped mineralized filaments on the oxidized layers (Figure 9D). The ubiquitous EPSs’ presence further indicates that indirect contact mechanisms dominate microbe–mineral interaction on the Ccp.

**Figure 16:**
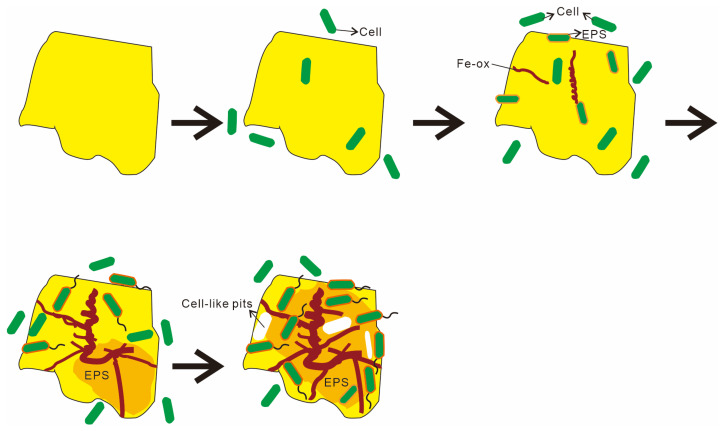
Sketches of the microbial weathering process of hydrothermal sulfides on the seafloor.

Based on microscopic observations and changes in the organic functional groups, this study outlines four distinct stages of microbe–mineral interactions (Figure 16). Stage 1 describes the initial approach. Microbes approach the mineral surface through diffusion, sedimentation, and cell motility (Figure 8A,B). During this phase, bacteria secrete EPSs, and macromolecular groups within the EPS interact specifically with the mineral substrate. The increased relative abundance of aliphatic and aromatic groups (Figure 14 and Figure 15) indicates hydrophobic bond formation, enhancing the mineral surface hydrophobicity [81]. Stage 2 describes the initial adsorption. Negatively charged bacteria adsorb to positively charged mineral surfaces via electrostatic attraction [82], as shown in Figure 8C,D. Stage 3 comprises stable adsorption, in which the microbial cells firmly adhere to the mineral surface, making detachment difficult. Bacteria secrete EPSs rich in lipopolysaccharides and proteins (Figure 8F,G), supported by increased polysaccharide and aliphatic components on Py (pyrite)-dominated surfaces (Figure 14). Additionally, the presence of bacterial appendages such as pili (Figure 9B) further strengthens the interactions between the bacteria and the mineral surface, helping the cells adhere more stably to the sulfide surface [83]. Stage 4 comprises extensive colonization. This stage is characterized by extensive microbial colonization on the sulfide surface, particularly in Ccp-dominated samples. Bacteria proliferate on the mineral surface and within biofilms, resulting in the formation of numerous new cells (Figure 9D). Microbial cells directly interact with the sulfide surface, forming cell-like dissolution pits (Figure 9D,E). In Ccp-dominated samples, a significant increase in aliphatic and phosphate organic components also reflects the higher microbial activity [37].

It should be noted that the influence of environmental factors (e.g., pH, temperature gradients, and salinity) on microbial colonization stages remains an open question. Future studies integrating real-time environmental monitoring and controlled laboratory simulations are essential to resolve these interactions.

## 5. Conclusions

Our 18-month in situ incubation experiment at the Wocan-1 hydrothermal field elucidates the distinct microbial weathering mechanisms governing Py and Ccp in deep-sea environments, with critical implications for marine biogeochemical cycles and ecological risk assessments. The key findings are summarized as follows:

1. Microbial influence on seafloor sulfide weathering. Microorganisms play a crucial role in the weathering of seafloor sulfides. Both the Py and Ccp samples were influenced by the combined effects of chemical and microbial weathering.
2. Differences in microbial weathering mechanisms. The microbial weathering mechanisms for the two sulfides differ significantly. The microbial weathering of Py is primarily mediated by an EPS-driven indirect contact mechanism, whereas Ccp weathering results from the interplay of both direct and indirect contact mechanisms.
3. Distinct stages of microbe–mineral interaction. The process of microbe–sulfide mineral interaction can be summarized in four phases: (1) microbes approach the mineral surface via diffusion, sedimentation, and cell motility; (2) negatively charged bacteria adsorb onto positively charged mineral surfaces through electrostatic attraction; (3) microbial cells firmly adhere to the mineral surface, making detachment difficult; (4) extensive colonization of the mineral surface by microbial cells occurs, leading to biofilm formation and sustained weathering activity.

This study advances our understanding of microbial weathering processes and the mechanisms of various sulfide minerals in the seafloor environment. It highlights the impact of microbial activity on marine biogeochemical cycles and provides a foundation for exploring the potential effects of microbe–sulfide interactions on marine ecosystems. Furthermore, the findings offer insights into sustainable deep-sea mining practices by offering guidance on mitigating the ecological risks associated with sulfide mineral exploitation.

## Figures and Tables

**Table 1:** Sulfide samples, as selected for the seafloor in situ incubation experiments (microscopic observations).

Type	Sample Description
Py-dominated	Pyrite-dominated, with a porous texture
Ccp-dominated	Chalcopyrite-dominated, minor pyrite and sphalerite, well crystallized

**Table 2:** Major FT-IR absorption bands.

Wave Numbers (cm)^−1^	Vibration Types	Functional Groups	Reference
2850~3000. 1465, 1451, 1444	C-H stretching vibrations (νC-H), corresponding to CH_3_ and CH_2_;	Aliphatic compounds	[23,38]
1735, 1412, 1408, 1407, 1400, 1398, 1385, 1383, 1298	Vibrational C=O Stretching (νC=O)	Carboxylic acids	[23,39]
1660~1630	Stretching C=C; Stretching C=O	Amides	[23,24]
1610, 1509, 1507	C=C;	Aromatic compounds	[38,40,41,42]
1270–1220, 1100–1070, 1000–950	ν(P=O); ν(PO)^2−^	Phosphate	[23,38,43]
1200~950	Vibrations of C-OH, C-O-C, and C-C	Polysaccharides	[23,43]

**Table 3:** Relative intensity of characteristic peaks before and after in situ incubation.

Sulfide	Treatment	Aliphatic Compound	Carboxylic Acids	Amides	Aromatic Compound	Phosphate	Polysaccharide
Py	pre-incubation	0	4.25%	13.1%	0	36.82%	45.92%
	post-incubation	1.37%	3.28%	9.66%	0.34%	23.11%	62.23%
Ccp	pre-incubation	6.96%	5.36%	21.16%	0	0	66.52%
	post-incubation	24.71%	12.92%	22.21%	11.21%	6.12%	22.83%

## Data Availability

The original contributions presented in this study are included in the article. Further inquiries can be directed to the corresponding author.
